# Idiopathic Anaphylaxis

**DOI:** 10.1007/s40521-017-0136-2

**Published:** 2017-06-03

**Authors:** Bright I. Nwaru, Sangeeta Dhami, Aziz Sheikh

**Affiliations:** 1 Allergy and Respiratory Research Group, Usher Institute of Population Health Sciences and Informatics, The University of Edinburgh, Medical School Doorway 3, Teviot Place, Edinburgh, EH8 9AG UK; 2 Evidence Based Healthcare Ltd, Edinburgh, UK

**Keywords:** Anaphylaxis, Corticosteroids, Diagnosis, Epinephrine, H1-antihistamine, Treatment

## Abstract

Idiopathic anaphylaxis is a rare life-threatening disorder with symptoms similar to other forms of anaphylaxis. There is lack of a robust evidence base underpinning the treatment of anaphylaxis and even less so for idiopathic anaphylaxis. Much of the evidence therefore comes from relatively small case series and expert opinion. Idiopathic anaphylaxis is a diagnosis of exclusion, requiring a thorough history and careful diagnostic work-up investigating possible triggers and underlying predisposing factors. Key diagnostic tests include skin-prick testing, tests for specific-IgE, component-resolved diagnostics, and in some cases for allergen challenge tests. Other recognized causes of anaphylaxis, such as foods, medications, insect stings, latex, and exercise, should all be considered, as should differential diagnoses such as asthma. While the cause of idiopathic anaphylaxis remains unknown, prompt treatment with intramuscular epinephrine (adrenaline) administered into the anterolateral aspect of the thigh is associated with good prognosis. There may also be a role for H1-antihistamines and corticosteroids as second-line agents. Patients need to be carefully monitored for signs of deterioration and/or a possible protracted or biphasic reaction. Patients with frequent episodes of anaphylaxis (e.g., six or more episodes/year) should be considered for preventive therapy, which may include corticosteroids, H1- and H2-antihistamines, and, in some cases, mast cell stabilizers such as ketotifen. Alternative immune-suppressants (e.g., methotrexate) and anti-IgE may rarely also need to be considered. In many cases, the frequency of anaphylaxis declines such that regular use of corticosteroids can be discontinued after 9–12 months. Pediatric patients should be treated with similar regimens as adults, but with appropriate dose adjustments. Patients should carry their self-injectable epinephrine and other emergency medications at all times in order to deal with emergency situations.

## Introduction

Anaphylaxis has been defined as a “…serious life-threatening generalized or systemic hypersensitivity reaction,” which is typically rapid in onset and associated with skin and mucosal changes; it may in some cases prove fatal [1••, 2••, 3••, 4••]. Idiopathic anaphylaxis is a rare form of anaphylaxis for which triggers cannot be identified despite a detailed history and careful diagnostic assessment [5••, 6••, 7–9]. Its manifestations are identical to other forms of anaphylaxis [5••, 6••, 7–9]. Idiopathic anaphylaxis was first described by Bacal and colleagues in 1978 in a report of 11 patients whose episodes could not be explained [10••]. Although the actual incidence and prevalence of idiopathic anaphylaxis are difficult to estimate, it has been suggested that as of 1995 in the USA alone, there were, since it was first described, at least 50,000 patients with idiopathic anaphylaxis [6••, 11••, 12]. According to most reports, idiopathic anaphylaxis appears to be more common in females than in males, with several studies indicating that over 60% of cases are females [13, 14]. While idiopathic anaphylaxis was first reported amongst adults, subsequent reports show that it is a condition that may affect people of all ages, although it is more common in adults than in children [15–18].

### Diagnosis

As the name indicates, the etiology of idiopathic anaphylaxis remains uncertain—it is therefore a diagnosis of exclusion [5••, 6••, 7–9]. The diagnosis should only be made after a very thorough clinical history, physical examination, and diagnostic testing to exclude both possible triggers for anaphylaxis and possible differential diagnoses [1••, 2••, 3••, 4••, 5••, 6••, 7–9]. Food is the most common trigger of anaphylaxis, accounting for up to 30% of fatal cases [1••, 2••, 3••, 4••]. The most common foods implicated include cow’s milk, egg, soy, peanuts, tree nuts, fish, and shellfish [1••, 2••, 3••, 4••]. In addition, other uncommon, so-called hidden food allergens should be considered as a differential diagnosis. An example of this is the so-called pancake anaphylactic syndrome or oral mite anaphylaxis, which is a new syndrome that presents with severe allergic symptoms and manifests after ingestion of foods made with mite-contaminated wheat flour, particularly pancakes [19, 20].

Other known causes, such as medications, insect stings, latex, and exercise, should be considered as should a host of other differential diagnoses of anaphylaxis [1••, 2••, 3••, 4••]. In exercise-induced anaphylaxis, the immediate trigger of symptoms is exercise, but in some patients, symptoms only occur if exercise occurs after a few hours of ingesting a specific food; this is known as food-dependent, exercise-induced anaphylaxis [3••, 21, 22]. The search for triggers typically includes skin-prick testing and tests of specific-IgE [1••, 2••, 3••, 4••] and may also include component-resolved diagnostics [22] and challenge testing. Galactose-alpha-1,3-galactose should be considered, particularly if there is a history of red meat ingestion several hours before the attack [23]. Serum tryptase levels can be very helpful in confirming the diagnosis of anaphylaxis. Systemic disorders, which may present with symptoms akin to anaphylaxis, also need to be judiciously excluded, including causes such as carcinoid syndrome, mastocytosis and mast cell activation syndromes (MCAS), and pheochromocytoma [1••, 2••, 3••, 4••]. Mast cell-derived mediators can, for example, be helpful if considering the diagnosis of MCAS [24], and C4 (and in some cases C1inh) concentrations can be helpful if considering a diagnosis of hereditary angioedema [25].

### Clinical features and classification

Idiopathic anaphylaxis presents clinically as anaphylaxis of unknown etiology [1••, 2••, 3••, 4••, 26••]. It is a multi-system disorder that may present with a number of symptoms and signs including hives, urticaria, diarrhea, vomiting, and angioedema; crucial to the diagnosis of anaphylaxis however is the presence of respiratory and/or cardiovascular compromise, which may present as cough, wheeze, dyspnea and tachycardia, light-headedness, and shock [1••, 2••, 3••, 4••]. Idiopathic anaphylaxis can be classified according to the frequency and manifestations of attacks [5••, 6••, 7–9]. For example, patients experiencing six or more episodes per year or two or more episodes in 2 months may be classified as idiopathic anaphylaxis-frequent, while those experiencing fewer episodes can be classified as idiopathic anaphylaxis-infrequent [5••, 6••, 7–9]. Classifications by manifestations of symptoms are idiopathic anaphylaxis-generalized and idiopathic anaphylaxis-angioedema [5••, 6••, 7–9, 11••]. Patients with the generalized form may experience symptoms of hypotension, gastrointestinal upset, bronchospasm, or cardiovascular collapse, whereas anaphylaxis-angioedema patients experience angioedema or urticaria with upper airway compromise (resulting from laryngeal, pharyngeal, and/or tongue edema) [5••, 6••, 7–9, 11••]. These two classification approaches are combined into four categories to form a summary diagnosis: idiopathic anaphylaxis-generalized-frequent, idiopathic anaphylaxis-generalized-infrequent, idiopathic anaphylaxis-angioedema-frequent, and idiopathic anaphylaxis-angioedema-frequent [5••, 6••, 7–9, 11••].

## Treatment

Despite the uncertainty of its etiology, idiopathic anaphylaxis is now a well-recognized entity which, if appropriately treated, is associated with a good prognosis [5••, 6••, 7–9, 26••]. For acute attacks, intramuscular epinephrine in a dose of 0.01 mg/kg of a 1:1000 (1 mg/mL) solution to a maximum of 0.5 mg in adults and 0.3 mg in children [1••, 2••] is the first-line agent. This is followed by provision of emergency care for patients for continued treatment and monitoring [5••, 6••, 7, 9, 11••]. There may also be a role for corticosteroids and H1-antihistamines, which can be given either orally or parenterally [1••, 2••, 3••, 4••, 5••, 6••, 7–9, 26••].

All patients with a diagnosis of idiopathic anaphylaxis should be instructed in the management of an acute episode. This primarily involves early recognition of the condition and the prompt use of epinephrine. Patients/carers should be prescribed and instructed in the use of an epinephrine auto-injector and encouraged to carry it with them at all times along with an H1-antihistamine and corticosteroids [1••, 2••, 3••, 4••]. This advice is best communicated through a written and/or electronic self-management plan [27].

Longer-term management of patients with idiopathic anaphylaxis is primarily determined by the frequency and severity of episodes and is initiated on an individual basis. There are no standard treatment regimens for this condition and limited robust research has been conducted. Treatments are based on case series, observations, and expert opinion. The algorithm developed by Patterson [26••] and colleagues and subsequently enhanced by others [6••, 7, 28••] has proven useful for the management of idiopathic anaphylaxis. Patients with frequent episodes require maintenance therapy, which includes 40–60 mg daily prednisone and an H1-antihistamine in the form of 10 mg cetirizine, 25–50 mg hydroxyzine, 25–50 mg diphenhydramine, or 180 mg fexofenadine [5••, 6••, 7, 9, 11••, 26••]. Steroids are typically not required in those with less frequent anaphylaxis attacks.

### Pharmacologic treatment

Classical pharmacologic treatment options for the management of idiopathic anaphylaxis include epinephrine, H1-antihistamines, and glucocorticoids; each will be considered in turn below [5••, 6••, 7, 9, 11••]. Other treatments sometimes used include H2-antihistamines, mast cell stabilizers, anti-leukotrienes, and anti-IgE. In clinical practice, these treatments may be used ahead of regular use of steroids to minimize the side effects associated with long-term use of systemic steroids.

#### Epinephrine

Epinephrine is the first-line treatment option used for the management of acute anaphylactic attacks [1••, 2••, 3••, 4••]. It is potentially life-saving given that it has both alpha- and beta-adrenergic vasoconstrictor effects in most body organs and is able to both relieve airway obstruction resulting from mucosal edema and reverse hypotension/shock through its ionotropic and chronotropic effects [1••, 2••, 3••, 4••]. Further, epinephrine decreases mediators released from the mast cells and basophils [29]. Epinephrine should be promptly administered once symptoms of anaphylaxis are confirmed [1••, 2••, 3••, 4••]. It should be injected intramuscularly at a dose of 0.01 mg/kg of a 1:1000 (1 mg/mL) solution, to a maximum of 0.5 mg in adults and 0.3 mg in children, into the anterolateral aspect of the thigh. This should be repeated every 5–15 min, if necessary; most patients respond after one or two doses [1••, 2••, 3••, 4••, 5••, 6••, 7, 9, 11••]. It is generally recommended that epinephrine should be administered in a supine position unless there is respiratory compromise when the patient should remain seated [1••, 2••, 3••, 4••, 11••].

Training devices are readily available and online videos can be used to instruct patients on how to use their auto-injector (e.g., www.youtube.com/watch?v=tjILFYPE3Uw; www.youtube.com/watch?v=HF5a2j7mHr8). Emergency medications should be regularly checked to ensure they have not expired [1••, 2••, 3••, 4••]. Patients/carers may benefit from an anaphylaxis management plan which can also be shared with school, work, etc. [30] with clear written instructions of how to manage an acute attack [1••, 2••, 3••, 4••]. It is important to clearly record the diagnosis in the patient’s notes and wearing emergency jewelry (e.g., a MedicAlert bracelet) should be encouraged [1••, 2••, 3••, 4••] so as to alert first-responders to administer epinephrine. Patients with idiopathic anaphylaxis may need special consideration and careful management of their condition during the perioperative period [1••, 2••, 3••, 4••]. Common side effects of epinephrine include dizziness, palpitations, and tachycardia [11••]. In some cases, failure to promptly administer epinephrine has been associated with an increased risk of particularly severe attacks and/or death [1••, 2••, 3••, 4••, 11••].

#### Glucocorticoids

The key glucocorticoid used for the treatment of idiopathic anaphylaxis is prednisone [5••, 6••, 7, 9, 11••]. Prednisone (40–60 mg) is often used following the administration of epinephrine to treat an attack [5••, 6••, 7, 9, 11••]. For those with frequent attacks, a maintenance therapy is recommended at a dose of 40–60 mg daily for at least 1 week or until symptoms subside [5••, 6••, 7, 9, 11••]. Once symptoms are controlled, alternate-day intake of prednisone at the same dose that controlled the symptoms is given and the dose is decreased by 5–10 mg each month [5••, 6••, 7, 9, 11••]. With this regimen, most patients discontinue prednisone after 9–12 months, and studies indicate that this treatment substantially decreases the frequency and recurrent life-threatening episodes in idiopathic anaphylaxis patients [5••]. After discontinuation of prednisone, patients may require to continue daily H1-antihistamines [5••, 6••, 7, 9, 11••, 13]. About 20% of patients with frequent idiopathic anaphylaxis may fail to wean from prednisone; these are thus considered as corticosteroid-dependent idiopathic anaphylaxis [11••]. A prolonged regimen of prednisone is often required for these patients [5••, 6••, 7, 9, 11••]. Glucocorticoids are associated with long-term side effects, including type 2 diabetes, glaucoma, osteopenia, and psychosis. Caution should be exercised in using them in patients with diabetes, osteoporosis, and hypertension and elderly patients [11••]. Alternative treatments have been tried with some success in order to avoid the long-term use of steroids [1••, 2••, 3••, 4••, 5••, 6••, 7, 9, 11••]. These are discussed below.

#### H1- and H2-antihistamines

H1-antihistamines are often used in the management of acute attacks of anaphylaxis where they may help to relieve cutaneous symptoms. They may also be used in longer term as a preventive approach in those with idiopathic anaphylaxis [5••, 6••, 7, 9, 11••]. H1-antihistamines used for idiopathic anaphylaxis include the first-generation hydroxyzine 25–50 mg and diphenhydramine 25–50 mg and the second-generation fexofenadine 180 mg and cetirizine 10 mg [6••, 7, 11••]. When used, each of these H1 blockers is used once or twice daily in the stated doses. The second-generation H1 blockers are preferred to the first-generation types as second-generation H1 blockers allow for selective antagonism of peripheral histamine H1 receptors and do not cause sedation as do the first-generation H1 blockers [6••, 7, 11••]. Side effects associated with the use of H1-antihistamines may include diarrhea, dizziness, headaches, and dyspepsia [11••]. They should be used with caution in patients with renal insufficiency [6••, 7, 11••]. Caution should also be exercised to ensure that sedating H1-antihistamines are not used [31]. For long-term management, it is recommended to use a combined regimen of steroids and H1-antihistamines and there is evidence that up to 50% of patients go into remission after completing the recommended regimen [6••, 7, 11••]. Patients whose symptoms are not controlled with an H1-antihistamine may benefit from addition of H2-antihistamines [32].

### Emerging therapies

#### Omalizumab

Omalizumab is a humanized monoclonal antibody that recognizes and binds to the Fc portion of the IgE molecule, thus blocking IgE binding to mast cells and basophils [33–36]. It is a recognized treatment option for chronic, moderate, and severe persistent allergic asthma [37] and has also been shown to play important therapeutic role in other IgE-mediated conditions such as seasonal and perennial allergic rhinitis and chronic idiopathic urticaria [38–40]. Recently, in a series of case studies, omalizumab has been used to treat idiopathic anaphylaxis [33–36]. Idiopathic anaphylactic patients administered 300–375 mg dose of omalizumab once every 2 to 4 weeks, after corticosteroid and antihistamine treatments did not reduce the frequency of attacks, were found to be without any anaphylactic episodes in about 6–12 months of using omalizumab [33–36]. This is however an emerging therapy in the context of idiopathic anaphylaxis as it has only been demonstrated in a few cases. Omalizumab is a very expensive treatment regimen and there are current concerns that it can also in some cases cause anaphylaxis; therefore, patients receiving it require a period of observation after the injections [11••].

#### Other agents

Ketotifen, an H1-antihstiamine and mast cell stabilizer, has been shown to be effective in reducing or terminating the dose of prednisone in corticosteroid-dependent idiopathic anaphylaxis patients, although it has been shown to also cause severe sedation [6••, 11••, 41] and weight increase in children [42]. Other second-line alternatives that may be steroid-sparing include anti-leukotrienes and oral cromolyn or oral albuterol [1••, 2••, 3••, 4••, 11••].

### Pediatric considerations

Pediatric idiopathic anaphylactic patients should be classified and treated with similar regimens as adults but with dose adjustments for steroids, epinephrine, and antihistamines [2••]. Omalizumab is licensed for use in those 12 years and older [33–36].

## Conclusions

Since it was first described 40 years ago, it has been possible to identify the trigger factor in a small proportion of those who previously would have been diagnosed as having idiopathic anaphylaxis. It remains a diagnosis of exclusion, which if appropriately treated is associated with a good prognosis. Self-injectable epinephrine should be promptly administered intramuscularly and plans should then be made to transfer patients to an emergency department for monitoring. Repeated doses of epinephrine can be administered every 5–15 min, if necessary. Preventive approaches for those with frequent episodes include regular use of prednisone and non-sedating H1-antihistamines. Ketotifen, anti-leukotrienes, and omalizumab may all need to be considered to reduce the frequency of attacks and/or reduce the steroid load. Similar regimens used for treatment of idiopathic anaphylaxis in adults can also be used in the pediatric population although with appropriate adjustments of the doses of steroids, epinephrine, and antihistamines. It is hoped that further advances into the epidemiology and mechanisms of anaphylaxis will help to reduce the numbers of patients labelled as having idiopathic anaphylaxis.
